# Standards adoption: A comprehensive multidisciplinary review

**DOI:** 10.1016/j.heliyon.2023.e19203

**Published:** 2023-08-16

**Authors:** Geerten van de Kaa

**Affiliations:** Faculty of Technology, Policy, and Management, Delft University of Technology, Jaffalaan 5, 2628BX, Delft, the Netherlands

**Keywords:** Standards adoption, Standardization, Compatibility standards, Quality standards, Innovation adoption and diffusion, Innovation management

## Abstract

The paper provides an overview of determinants for the adoption of standards; a topic on which little research has been done so far. An extensive review and systematic analysis was conducted of the papers that have published on the topic. This resulted in a framework with 18 factors for the adoption of standards divided into 5 categories. A distinction is made between factors for the adoption of compatibility standards and quality standards. Additional analysis have been performed investigating the completeness of the list of factors and the extent to which cross-fertilization occurs by authors that study the topic of standards adoption. The paper concludes with contributions, limitations and a future research agenda.

## 1 Introduction

The process of standardization can be divided into four stages; development and design, acceptance and enforcement, choice and diffusion, and impacts [1]. Plenty of standardization researchers have studied standard development and design [2,3]. They focus e.g. on how standards are developed [[4], [5], [6], [7]] and the reasons why companies engage in the standardization process [[8], [9], [10]]. Other researchers have focused on the process of standards diffusion and have looked at specific factors for standard dominance [[11], [12], [13]]. These scholars have studied standards battles [[14], [15], [16], [17], [18], [19], [20], [21], [22], [23]] and have identified factors that can be influenced by firms in order to achieve standards selection [24,25]. Finally, scholars have studied the economic effects of standards [26] and have focused on the impact that standards may have [27] on, e.g., innovation [28].

However, in these studies, the firm that has to make a decision to adopt a standard is often seen as a black box. Few scholars have focused on the process of standards choice (or adoption) [29]. And when they do, they take the perspective of the standards organization^1^ and focus on how that entity can make sure that the standard will be adopted by the firm [30]. One exception is internet standards adoption which has been studied by a handful of researchers [[31], [32], [33]]. However, one unified framework that integrates determinants for standards adoption by companies is missing. This paper has as a goal to realize such a framework and raises the question: which factors affect the chances that companies adopt standards?

A systematic literature review has been conducted which resulted in a total of 18 factors for standards adoption divided into 5 categories. We analyse the extent to which cross-fertilization in the field of standards adoption can be observed, the completeness of the list of factors and the extent to which factors for standards adoption depend on the type of standard.

Standardization scholars distinguish between safety standards, variety-reducing standards, information and measurement standards, compatibility (or interface) standards, and quality standards [26]. In this paper, we focus on the latter two. Compatibility standards ‘define the interface between two or more mating elements that are compatible rather than similar’ [34]. An example includes the universal serial bus (USB) standard that defines communication between, amongst others, the personal computer and its peripheral equipment. A quality standard ‘specifies properties of a material object that are essential for its use and specifies related assessment criteria.’ [35] An example includes the ISO 9001 standard that specifies how a firm's quality management system should be set up. So where compatibility standards ensure interoperability of components within a system, quality standards define how an object (such as a system) should be set up in order to function properly.

This study contributes to the literature on standardization [26,36,37] by, for the first time, developing a general framework for factors for standards adoption. Scholars can apply the framework to explain and, possibly, predict standards adoption. Practitioners, such as companies, can use this framework as a checklist to assess whether to adopt the specific standard under consideration. Governments and standards organizations may use the checklist to devise a strategy how to ensure that companies adopt their standards.

## 2 Theoretical background

Several researchers from a diverse range of perspectives have looked at how standards are established. Evolutionary economists argue that technology advancement is characterized by sudden events that every once in a while shake up a sector considerably [38]. These technological discontinuities act as a forebode for radical technological innovation whereby a set of path-dependent choices converge to a single dominant design and de facto standard [39,40]. This theoretical stream of literature propagates that technology development occurs cyclical [41] and that a dominant design or standard is the result of a set of choices that cannot be predicted in advance. Strictly following these logics, standards adoption cannot be determined ex ante but only ex post and, therefore, standards adoption factors cannot be determined.

On the other hand, industrial economists argue that standards become adopted as a result of economic mechanisms. In markets that are characterized by increasing returns to adoption the value of a technology to users increases as it becomes more adopted. Technology that gains an early lead can dominate the market [42]. The sources underlying the returns include network effects [43,44]. Because of these economic effects, users often follow each other in their adoption choices because the expected benefits of the standard increases, possibly, resulting in a bandwagon effect [44]. In markets characterized by network effects, switching costs, may, amongst others, influence the adoption of standards [45]. These costs may become so high it becomes difficult for users to adopt another standard and they are locked into the standard [46]. A good example of where such a phenomenon occurred is the standard that defines the arrangement of letters on keyboards; QWERTY [47]. Although by utilizing these insights, standards adoption can be explained, extant research in this literature stream primarily focuses on standards adoption by consumers and not by companies.

Scholars that focus on standards adoption by companies have argued that the reasons why companies adopt a particular standard, in part, stem from forces acting on the firm. Using institutional theory, they often cite existence of normative, mimetic, and coercive pressures [48] that act on the firm and potentially cause it to choose a particular standard [49]. For example, suppliers that cooperate with buyers in transactional or relational contracts will have a higher chance to adopt financial reporting standards [50]. And when voluntary standards are supported by a regulator, the chances are higher that they will be adopted by companies [51]. Normative pressures also may lead to standards adoption; Mzembe [52] showed that a hotel's adoption of sustainability standards is, in part, dependent upon its own prior experience with sustainability.

Various scholars apply innovation adoption and diffusion literature [53,54] to explain why companies adopt standards [[55], [56], [57]]. These scholars posit that the characteristics of the standard itself influence whether it will be adopted [32,33]. They also argue that the extent to which the standard is compatible with the firm will affect whether the firm will adopt the standard. For example, Dos Santos and Reinhard [58] argues that compatibility standards adoption in Brazil may be negatively affected by the lack of resources of the firm required for implementing the standards.

Scholars that have a background in innovation management and standardization have investigated why dominant designs and standards are established [25,[59], [60], [61]]. They argue that the characteristics and strategies of standards organizations may influence the chances that standards achieve dominance [12,62,63].

The last decades, scholars have studied factors that affect the emergence of single dominant designs such as the level of openness of the design [64] and timing of entry strategies [65]. Few of these scholars focus on the factors that affect the chances that firms adopt standards. These scholars primarily focus on what standards organizations can do to achieve success with their technological standard. The few scholars that do specifically focus on the topic of standards adoption conduct mostly qualitative empirical research discussing specific factors for standards adoption. They often combine two or more of the theoretical approaches discussed above. For example, Hovav et al. [32] focuses on the adoption of the IPv6 internet standard. They suggest that two categories of factors related to the characteristics of the technology and the context in which standards adoption takes place, affect whether and how these standards are adopted. Other scholars have conducted case studies in which standards adoption is studied. Kedzior [66] focus on which factors affect the adoption of financial reporting standards in Poland. They empirically study the effect of firm size and the (international) ownership of the firm on the chances that the firm will adopt the standard. Table 1 presents the four research streams discussed above, their view towards standards adoption, and the type of factors for standards adoption on which they focus.

**Table 1:** Main theories and approaches utilized by researchers studying standards adoption.

Literature stream/theoretical approach	Category	Factors for standards adoption	Explanation
Network economics [43,44]	Market mechanisms	Network externalities and switching costs	When more firms adopt a certain standard, the value of that standard increases and it becomes more beneficial to choose that standard over other standards.
Neo-institutional theory [48,67]	Pressures	Normative, mimetic, and coercive pressures	A standard is adopted due to pressures that act upon the firm. This pressure can come from the firm or from other actors.
Innovation adoption and diffusion [53,54]	Standards characteristics	Relative advantage and comprehensibility	The choice to adopt a standard depends on its technical characteristics and the extent to which it is compatible with the firm.
Innovation management and standardization [62]	Characteristics of the firm; Standards organization's characteristics and strategies	firm size and standard's price	The choice to adopt a standard depends on the characteristics of the firm that adopts it and the standards organization that offers it (and its strategies).

## 3 Methods

In order to give an answer to the question what factors affect the chances that standards are adopted by firms a systematic literature study was conducted utilizing the ISI web of knowledge. The topic “standards adoption” was searched for in the title, abstract, author keywords and keywords plus. This resulted in 121 hits. The abstracts of these 121 papers were read. Papers were studied in detail if they presented empirical studies of factors influencing the *adoption* of standards by *firms*. So papers that studied the effect of standards adoption on, e.g., the financial performance of firms [68] were not included. Also, papers that studied the adoption of standards by governmental organizations [69] or countries [70] were not included. For each paper we gathered data on the type of standard that is investigated so that it is possible to investigate whether the relevance of factors for standards adoption is dependent upon that.

**Fig. 1:**
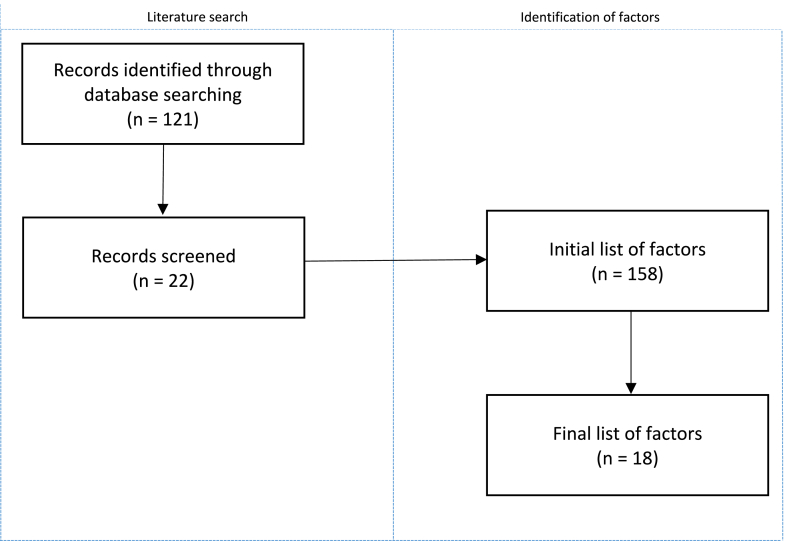
Flowchart of the intermediate steps and results of the analysis.

Twenty-two papers were found. Each paper was screened for factors of standards adoption. Criteria used to screen the articles include; (1) the factor for standards adoption should explicitly be mentioned, (2) the standard discussed in the paper should be either a compatibility standard or a quality standard. When a factor was mentioned in an article, it was added to an initial list of factors. The initial search resulted in 158 factors. In a second analysis, this initial list of factors was shortened into a list of 18 factors by looking for similarities and by combining factors that refer to a similar aspect or that were similar but mentioned under different names. In a third analysis, based upon their similarity, the factors were categorized into the 5 categories mentioned in section 2. This is a proven approach for conducting similar studies into, e.g., factors for standards dominance [25]. The complete process is graphically illustrated in a flowchart (see Fig. 1).

## 4 Results

The analysed papers (available upon request) were published in diverse journals in the fields of Business & Economics (e.g. academy of management journal and research policy); computer science (e.g. information systems journal), and environmental sciences (journal of cleaner production). Scholars mostly utilize insights from the areas of technology adoption and/or network economics and some papers utilized insights from the institutional theory to explain standards adoption. There are also other theoretical perspectives that are incidentally utilized such as social network theory and transaction cost theory. This shows that, comparable to studies that focus on factors affecting standards dominance [25], the topic of standards adoption seems to be approached in a multidisciplinary manner by researchers.

From a methodological point of view, there is also diversity. For example, both case study research and simulation methods are used. Modes of observation in the papers include survey research (where data is analysed through applying e.g. regression analysis and structural equation modelling), qualitative field research (utilizing, e.g., qualitative interviews), and unobtrusive measures (through, e.g., content analysis).

A table which indicates which factor is mentioned in which article and their direction is included in Table 2. The table makes a distinction between factors for compatibility standards adoption and quality standards adoption so that it is possible to investigate whether relevant factors for standards adoption differ depending upon the type of standard.

**Table 2:** Detailed results.

	Direction	Chen [55]	Hovav, et al. [32]	Hovav and Schuff [33]	Chong and Ooi [56]	Hovav, et al. [31]	Henderson, et al. [49]	Techatassanasoontorn and Suo [45]	Xu, et al. [71]	dos Santos and Reinhard [58]	Alkraiji, et al. [72]	Xu, et al. [73]	Alkraiji, et al. [74]	Henning [75]	Ezingeard and Birchall e [76]	Zhou, et al. [77]	Moratis and Widjaja [78]	Wang, et al. [57]	Labella, et al. [79]	York, et al. [51]	Jajja, et al. [50]	Kedzior, et al. [66]	Mzembe, et al. [52]
Category/factor		Compatibility standard													Quality standard								
A. Standards characteristics																							
A.1 Technological superiority	+		X					X						X									
A.2 Relative advantage	+	X	X										X	X	X	X	X						
A.3 Observability	+	X	X	X																			
A.4 Comprehensibility	+		X								X						X						
A.5 Customizability	+													X			X						
B. Environmental factors																							
B.1 Network externalities	+		X				X	X					X										
B.2 Switching costs	–		X					X			X		X										
C. Pressures																							
C.1 normative pressures	+								X		X		X			X	X						X
C.2 mimetic pressures	+						X		X			X					X						
C.3. coercive pressures	+		X	X	X	X	X		X	X	X	X	X	X	X	X	X	X		X	X	X	
D. Firm characteristics																							
D.1 commitment	+										X		X		X			X					X
D.2 firm size	+									X			X			X						X	X
D.3 organizational infrastructure	+									X	X		X	X			X						
D.4 learning orientation	+									X	X	X	X			X	X	X	X				
E. Characteristics and strategies of the standards organization																							
E.1. legitimacy	+										X						X						
E.2. stakeholder diversity	+																X	X					
E.3. communication	+	X	X				X										X	X					
E.4. price of standard	–			X				X															

A subset of the papers that have been analysed have conducted quantitative empirical research through e.g. surveys and report factors that are found to be statistically significant determinants of adoption. These factors are specified in Table 3 so that it is possible to distinguish between factors that may influence the adoption of standards and those that have been found to be statistically significant determinants of adoption. Furthermore, Table 4 reports the methodological details of each paper including the independent variables and confidence level, dependent variable, and type of statistical analysis.

**Table 3:** Statistically significant determinants of standards adoption.

	Compatibility standards	Quality standards
Category/factor		
A. Standards characteristics		
A.1 technological superiority		
A.2 Relative advantage		Zhou, et al. [77]
A.3 Observability		
A.4 comprehensibility		
A.5 customizability		
B.Market mechanisms		
B.1 network externalities	Henderson, et al. [2]	
B.2 switching costs		
C.Pressures		
C.1 normative pressures	Xu et al. [71]	Zhou, et al. [77]
C.2 mimetic pressures	Henderson, et al. [2]; Xu et al. [71]; Xu et al. [73]	
C3. coercive pressures	Chong et al. [56]; Henderson, et al. [2]; Hovav, et al. [31]; Xu et al. [71]; Xu et al. [73]	Zhou, et al. [77]; York et al. [51]; Jajja et al. [50]; Kedzior et al. [66]
D firm characteristics		
D.1 commitment		
D.2 firm size	Xu et al. [71]	Zhou, et al. [77]; Kedzior et al. [66]
D.3 organizational infrastructure		
D.4 learning orientation	Xu et al. [71]; Xu et al. [73]	Zhou, et al. [77]
E. characteristics and strategies of the standards organization		
F1. legitimacy		
F2. stakeholder diversity		
F3. Communication	Henderson, et al. [2]	
F4. price of standard

**Table 4:** Detailed overview of theory testing studies.

Study	Independent variables and confidence level	Dependent variable	Type of Statistical analysis
Zhou, et al. [77]	Firm size^a^ Relative advantage^c^ Coercive pressures^c^ Learning orientation^c^ Normative pressures^b^	Quality standards adoption	Ordinal logistic regression (N = 139)
Henderson, et al. [49]	Mimetic pressures^b^ Coercive pressures^b^ Network externalities^a^ Communication^b^	Compatibility standards adoption	Structural equation modelling; Partial Least Squares (N = 52)
Xu, et al. [71]	Normative pressures^c^ Mimetic pressures^b^ Coercive pressures	Compatibility standards adoption	Structural equation modelling (N = 544)
Xu et al. [73]	Mimetic pressures^a^ Coercive pressures^c^ Learning orientation^b^	Compatibility standards adoption	Structural equation modelling; Partial Least Squares (N = 194)
Chong et al. [56]	Coercive pressures^c^	Compatibility standards adoption	Logistic regression analysis (N = 109)
Hovav, et al. [31]	Coercive pressures^c^	Compatibility standards adoption	Ordinary least squares regression (N = 84)
York et al. [51]	Coercive pressures^b^	Quality standards adoption	Negative binomial regression (N = 5110)
Jajja et al. [50]	Coercive pressures^c^	Quality standards adoption	Logistic regression analysis (N = 164)
Kedzior et al. [66]	Coercive pressures^c^ Firm size^a^	Quality standards adoption	Logistic regression analysis (N = 445)

a Confidence level of 90%.

b Confidence level of 95%.

c Confidence level of 99%.; Confidence levels of full models are reported.

### 4.1 Standards characteristics

Standard characteristics include all the features of the standard that make it superior to other standards. This results in a higher chance that it will be adopted by firms. These characteristics include:

**Technological superiority:** this refers to the extent to which the standard is technologically superior to alternatives. Henning [75] refers to this concept as ‘maturity’; the extent to which technical problems or other uncertainties do not arise when the standard is implemented. Firms will choose to adopt the technologically more superior standard over other less technologically superior standards and the factor thus positively affects standards adoption.

**Relative advantage:** this refers to the extent to which benefits are realized when the standard would be adopted by the firm. For quality standards this relates to, e.g., the extent to which the standard will provide more structure in the firm [78] but this also relates to the extent to which implementation of the standard leads to a higher reputation, e.g., through achieving accreditation [76,77]. For compatibility standards it relates to the extent to which, e.g., system integration can be facilitated when the standard is implemented [74]. The standard that provides a higher relative advantage over other standards will be chosen by firms over other standards and this factor, thus, positively affects standards adoption.

**Observability** refers to the extent to which the standard is known by the firm; better understanding the benefits of adopting the standard will lead to reduced risks associated with its adoption [32] and a higher adoption rate among firms. When it is possible to try out the standard before it is implemented in the firm, observability can be improved [33]. Customers will opt for the standard that is more observable, and, that factor, therefore, positively influences standards adoption by firms.

**Comprehensibility** refers to the extent to which the standard is understood by the firm. For instance, Alkraiji et al. [72] found that in Saudi Arabia, people lacked understanding of the health care data standard which negatively affected its adoption. Also, according to Moratis and Widjadja [78] firms found the ISO 26000 standard too complex and more difficult to understand in comparison to other standards, negatively affecting its adoption - to better understand the standard, external consultants had to be hired. They also found the standard to be too broadly formulated; it included issues concerning child labour that do not apply for western countries such as the Netherlands. Firms will opt for more comprehensible standards over less comprehensible ones.

**Customizability** refers to the extent to which the standard can be easily adapted to the requirements of the firm. It also refers to the extent to which improvements are pro-actively made by the standards organizations. For example, the Blu-ray standard was deliberately modified in order to persuade firms to opt for that standard [80]. Firms prefer standards that can be customized to their requirements and therefore the higher a standard's customizability, the higher the chances that firms will opt for that standard instead of the more rigid standard. The factor therefore positively affects standards adoption.

### 4.2 Market mechanisms

Market mechanisms include factors that cannot be directly influenced by the firm and exist in the market and affect standards adoption.

**Network externalities** refer to the phenomenon whereby the (perceived) value of a standard increases for a firm as more firms support that standard [43,44]. It has a positive influence on standards adoption [29]. Network effects may be direct or indirect [81]. Direct network effects occur when products are interconnected while indirect network effects occur when complementary goods are available that increase the value of the core product. For example, the value of a smart phone to users increases when more other users adopt such a phone because users can connect to more other users (direct network effects). The value will also increase when there are more apps available to be used in conjunction with the phone (indirect network effects). When network externalities are present, firms will decide to adopt the standard that is chosen by more other firms.

**Switching costs** are the costs required to switch from an existing standard to a new standard. The higher these costs are, the lower the chance that the new standard will be adopted. For example, in a situation that an existing standard is applied in a firm and that firm has the choice to adopt a new standard, switching costs might apply. A firm might, e.g., lack the required personnel to apply the new standard, and, in that case, investments in new personal have to be made [72]. If a company has to switch from an existing standard to a new standard, then the company will choose the standard with lower switching costs.

### 4.3 Pressures

The internal or external influences that act on the firm. When pressures to adopt a standard are high this causes the firm to adopt a standard.

**Normative pressures** refer to the extent to which firms adopt a standard because they have an intrinsic motivation to do so. Employees in the firm might, e.g., have been taught about the standard in their education and now feel obliged to adopt it. For example, Zhou et al. [77] argue that when a firm's employees have access to training about food safety from, e.g., professional institutions or governmental agencies, the chances are higher that they will adopt such standards, because they more better see the need for it. Furthermore, in some cases employees in firms might have an intrinsic motivation to adopt standards to demonstrate social responsibility [78] or because they are driven by a sustainability agenda [52]. When companies are more intrinsically motivated to adopt a certain standard, they are more likely to choose to adopt that standard over others.

**Mimetic pressures** refer to the extent to which firms voluntarily follow the adoption choice of other actors. For example, a firm could follow a competitor to increase its credibility within the industry, possibly resulting in ‘herd behaviour’ where multiple firms make the same choice and follow the majority [71], e.g.. by adopting similar quality standards [78]. The reason for applying mimetic behaviour is also that firms are afraid of being perceived as less advanced than their competitors if they do not adopt similar (compatibility) standards [73]. When mimetic pressures are high, firms will choose to adopt the standard that is adopted by other firms.

**Coercive pressures** refer to the extent to which other actors directly or indirectly influence the firm to adopt the standard. These actors may include the government [58], competitors [33], customers [76], suppliers [58], partners [56], market intermediaries [51], entrepreneurs [51], or shareholders [66]. For example, the government may enforce the standard in which case adoption becomes mandatory [58]. The government may also lend subsidies for the adoption of the standard in the form of tax refunds [51], or it may set up awareness programs around the standard [32]. Pressures to adopt a standard may also emerge from competitors or customers who may be in the focal firm's network as well as outside of it [33]. Firms may, e.g., offer financial incentives for firms to influence them to choose for their standard [32]. Rumour had it, Warner was offered money to exclusively support Blu-ray [82]. Furthermore, for quality standards, customers may, e.g., expect that their suppliers are accredited [76,78]. When the coercive pressure to adopt a specific standard are higher, firms will choose to adopt that standard over other standards.

### 4.4 Firm's characteristics

This category refers to the characteristics of the firm that make them adopt the specific standard.

**Commitment** refers to the extent to which the firm attaches importance to standards. For example, the existence of a specific department focusing on standards is a sign of high commitment. When that is not implemented in a firm, that firm might ignore recommended standards and might therefore also refrain from archiving standards. Furthermore, it might not evaluate their implementation and it might not offer training on the use of standards. These are all signs of limited commitment to standards [57] which has a negative effect on the adoption of standards [83]. When firms are more committed to a specific standard, they will choose to adopt that standard over other standards.

**Firm size** relates to various dimensions including the firm's available financial resources, the number of employees in a firm, the extent to which it can benefit from economies of scale, a diverse workforce, and the extent to which it has negotiating power over suppliers [77]. For example, the financial resources can be used to purchase the standard and implement it in the firm. When firm size is higher, the firm will be more inclined to adopt a specific standard, and, firm size, therefore, affects standards adoption positively.

**Organizational infrastructure** refers to the extent to which in the firm an infrastructure is implemented that is compatible with the standard. This makes it more easy to adopt the standard [71]. For example, adopting a corporate social responsibility certificate is easier when a firm already has a quality management systems in place and when the quality standard ties in well with other quality standards that are used within the firm [78]. When a standard is more compatible with a firm's infrastructure, that firm will be more inclined to decide to adopt that standard over others.

**Learning orientation** refers to the extent to which the firm possesses the necessary expertise to apply the standard and the extent to which it is able to learn (from, e.g., other actors). Expertise relates to the extent to which ‘standardization talents’ [57] are present in the firm that can apply the standard - this has a positive effect on the chances that the standard is adopted by that firm [71]. Expertise also relates to experience with standards in general [78]. For example, Labella et al. [79] conducted a study among 330 firms in the olive oil production industry in Spain. They find that firms that previously have adopted an environmental management system standard (ISO 14001) will have a higher tendency to adopt a quality management system standard (ISO 9001). The more the firm has the required expertise to apply a specific standard, the more inclined it will be to adopt that standard over others.

### 4.5 Standards organization's characteristics and strategies

The fifth category concerns all characteristics and strategies of the standards organization that make the firm more willing to choose for the standard.

**Legitimacy** refers to the recognition that the standards organization has among firms (in terms of, e.g., international acceptance). A firm will be more inclined to decide to adopt a standard that is developed by a more recognized standards organization [78].

**Stakeholder diversity** refers to the extent to which relevant stakeholders are involved in the development of the standard which positively affects standards adoption [78]. Diversity refers to both the amount of industries that are represented in the standards organization [80] and the type of actors. For example, Wang [57] found that when both nongovernmental organizations for standardization and industry associations are involved in the development of a standard, this has a positive effect on the chances that the standard will be chosen by a firm. A firm will be more inclined to decide to adopt a standard that is developed by a more diverse set of stakeholders.

**Communication** refers to the extent to which the standard is promoted [55] and information about the standard is communicated to the adopting firm [32]. This can, e.g., be in the form of proper guidance offered by the standards organization with respect to the implementation of the standard [57,78]. The better the communication surrounding the standard, the higher the chance that it will be adopted.

**The Standard's price** refers to the price that is set by the standards organization [45]. The higher, the lower the chance that firms will choose to adopt the standard. For example, Hovav and Schuff [33] showed that firms preferred the IPv6 standard over the IPv4 standard because the latter was too expensive. The new standard IPv6 would reduce overall costs for the firm when it would be implemented. Firms will prefer to adopt cheaper standards over more expensive ones.

## 5 Discussion and conclusion

This paper has carried out an extensive literature review to arrive at a list of factors for standards adoption. This section presents additional analysis, contributions, implications, limitations and future research recommendations.

### 5.1 Additional analyses

**Fig. 2:**
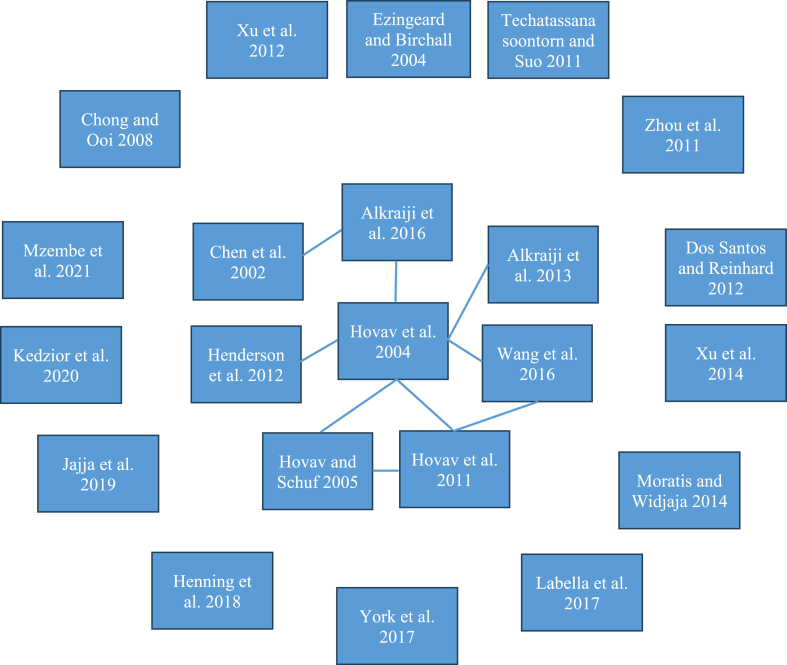
An analysis into the extent to which authors refer to each other.

Section 2 showed that authors utilize various theoretical perspectives and section 4 showed that standards adoption research has been published in diverse journals. The question is whether these authors make use of each other's results. To investigate this, a separate manual analysis was conducted into the extent to which authors in the dataset refer to each other (see Fig. 2). In Fig. 2, a rectangle stands for a paper. When a line is drawn between two boxes this means that the newer paper cites the older paper. Four things can be concluded. First, it appears that few scholars have focused on the topic of standards adoption. Second, Hovav et al. [[31], [32], [33]] were most active in this area. Third, some papers refer to one or more papers that were written by Hovav. Finally, most authors do not cite each other. Scholars may be doing similar research but may not be aware of that and, then, there is a potential risk that the cross fertilization of knowledge is low in this area.

**Fig. 3:**
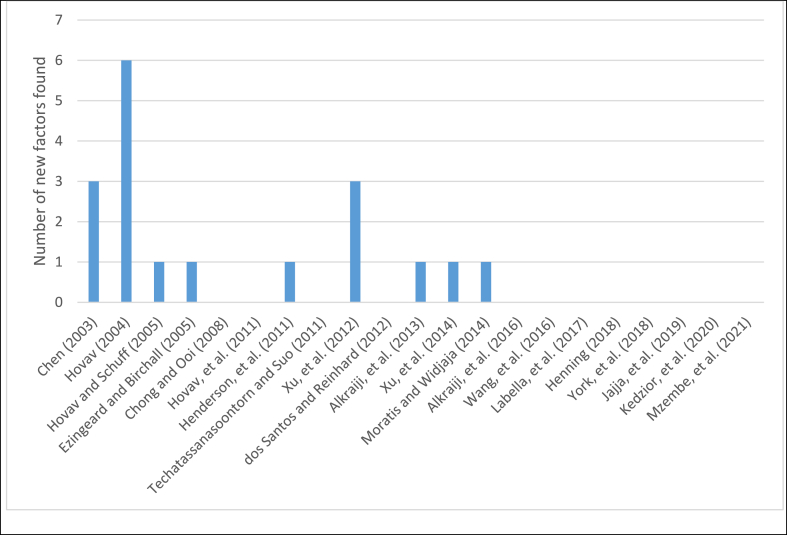
Number of new factors found per article.

To evaluate whether the list of factors is complete a separate analysis was conducted investigating the number of new factors found per article (see Fig. 3). It appears that in the most recent eight articles no new factors were found. This can be interpreted as a sign that the list is complete. Still, when future research would choose to investigate cases of standards adoption utilizing this paper's list of factors, it is recommended to keep the possibility open that new factors are added to that list.

From Table 2 we conclude that compatibility standards adoption appears to be influenced by all standards characteristics whereas quality standards adoption is influenced by all standards characteristics but technological superiority and observability. Furthermore, compatibility standards adoption is influenced by all market mechanisms whereas market mechanisms do not seem to influence quality standards adoption. All factors underlying pressures and firm characteristics seem to influence both compatibility standards adoption and quality standards adoption. Finally, compatibility standards adoption is influenced by all characteristics and strategies of the standards organization except stakeholder diversity. Quality standards adoption is influenced by all factors but the price of the standard.

### 5.2 Contributions and implications

This paper adds to the innovation management and standardization literature in various ways. First, it offers a framework with factors for standards adoption. By combining four streams of literature the paper conducts a multidisciplinary study thereby answering the call for more integrative research into standardization [84]. The framework identified 7 additional factors compared to the most complete framework in our dataset (which contained 11 factors). Therefore, it can be concluded that the framework is the most complete framework to date. Finally, the paper distinguishes between factors for the adoption of compatibility standards and quality standards.

Scholars can use the framework to explain the reasons why firms have chosen certain standards. They may also use the framework to predict which standard will be chosen by the firm. Various scholars that focus on the firm that offers a technology have shown that, contrary to what evolutionary economists believe, the process of standards selection can be modelled. By utilizing the current framework, scholars may also provide evidence that the process of standards adoption can be modelled and that factors can be identified. This can provide further evidence of the non-emergent character of standardization.

Firms can use the framework as a checklist. Often, multiple standards are available and a firm has to make a decision to adopt one of the standards. In this situation, the framework could be used to facilitate the decision making process. For example, a multi-criteria decision making process can be applied to assess the value of each of the alternative standards and make the best decision. Applying this method, the firm could, e.g., first evaluate which of the factors are relevant for the specific type of standard that is under investigation. Then, the importance of the relevant factors could be determined. Finally, the standards could be rated on each of the factors which results in the best standard to be chosen. This procedure could result in valuable input for the decision making process and potentially decrease the uncertainty attached to the decision to adopt one of the standards. We call for future research that attempts to conduct such studies as was previously also done for factors affecting standards dominance [[85], [86], [87]].

A similar approach can be followed by the standards organization that would like to increase adoption of their standard (when one or more alternative standards are available). The actor can use the checklist to estimate the chances that their standard and competing standards will be adopted and which factors affect that the most. It can then try to influence those factors. For example, let's assume that the outcome of such a study would be that technological superiority is one of the important factors and the favoured standard scores low on that aspect. Standards organizations may then investigate which technological aspects are not sufficient and try to improve those aspects.

The checklist may also be used by governmental agencies that hope to increase the adoption of a certain standard a similar approach as described earlier may be used. For example, let's assume that price of a standard is one of the most important factors and the favoured standard of the governmental agency is scoring low on that. In that case the price of the standard is too high resulting in customers adopting the less favourable standard. The government may then set up subsidies in an attempt to decrease the price and thereby incentive users to adopt the standard.

### 5.3 Limitations and recommendations

The papers that were investigated did not focus on safety standards and variety reduction standards and the factors, therefore, seem to be only applicable to compatibility standards or quality standards. Future research could focus on factors that influence the adoption for safety standards and variety reduction standards. Furthermore, further research could study the extent to which factors that influence the adoption for one type of standard (e.g. a compatibility standard) also influence the adoption for another type of standard (e.g. a quality standard). Also, future research could study whether the factors mentioned in this paper are related to each other. York et al. [51] provides some first evidence of this and other authors also hint at interrelations between factors. For example, Chen [55] argue that when the standard is promoted, awareness around the standard can increase which will increase the chances that the standard will be chosen.

Future research could also study the extent to which factors for standards adoption can be influenced. For example, the factor learning orientation can be influenced in various ways. The expertise needed to apply the standard can be improved by offering training activities [77]. Furthermore, firms can actively participate in events organized by actors and thereby learn from these actors as channels between these actors exist through which information about the standard is communicated [32]. It is argued that the more the firm learns through these channels from these actors, the more the advantages and effects of the standard will become known to the firm. This will decrease the uncertainty for firms that might exist around the standard [73]. It may also create positive expectations about the standard [32]. This will lead to an increase in the chances that the standard will be chosen by the firm. Future research can attempt to find out potential strategies which can be used to positively affect the other factors.

Finally, as can be concluded from Table 3, Table 4, most factors underlying the categories ‘pressures’ are found to be statistically significant determinants of adoption. Quantitative empirical research that studies factors for standards adoption in the other categories (the empty boxes in Table 3) is scarce. This provides a relevant area for future research; conducting quantitative empirical research into factors for the adoption of standards that, through qualitative research, have been shown to be relevant factors.

## Production notes

### Author contribution statement

All authors listed have significantly contributed to the development and the writing of this article.

### Data availability statement

No data was used for the research described in the article.

## Declaration of competing interest

The authors declare that they have no known competing financial interests or personal relationships that could have appeared to influence the work reported in this paper.

## Footnotes

1 A standards organization is a partnership, which develops and maintains a standard. Examples include standards consortia such as the USB implementors forum and standards development organizations such as ISO or IEEE.
